# Increased incidence of the type 1 diabetes and diabetic ketoacidosis severity in children during COVID-19 pandemic

**DOI:** 10.1186/s13098-024-01357-1

**Published:** 2024-05-31

**Authors:** Mohamad Ahangar Davoodi, Maryam Zamanian, Bahareh Balali

**Affiliations:** 1 Department of Pediatric Endocrinology, Clinical Research Development Center of Amirkabir Hospital, Arak University of Medical Sciences, P. O. Box 3819693345, Arak, Iran; 2 Department of Epidemiology, School of Health, Arak University of Medical Sciences, Arak, Iran; 3 Department of Clinical Nutrition, Master Student of Sports Physiology/Sports Nutrition, Arak University of Medical Sciences, Arak, Iran

**Keywords:** Type 1 diabetes (T1DM), Pediatric, COVID19 pandemic, COVID-19 peaks, DKA (diabetic ketoacidosis)

## Abstract

**Aim:**

The effect of COVID-19 on the occurrence of type 1 diabetes and ketoacidosis in children and adolescent.

**Methods:**

In this descriptive-analytical cross-sectional study, the records of all children and adolescents hospitalized due to type1 diabetes for two years ago and during the COVID-19 pandemic and its peaks were investigated (January 2018–2022). Also, the desired variables including the frequency of hospitalized patients (known and new cases), the frequency of DKA, the severity of DKA, the duration of discharge from DKA, age, body mass index, duration of hospitalization, clinical symptoms including cerebral edema, laboratory data and the total daily dose insulin required at the time of discharge were compared and statistically analyzed.

**Results:**

Out of the 334 hospitalized T1DM patients, the rate of new T1DM patients was significantly higher (*P* = 0.006) during the pandemic. Clearly, there were more cases of DKA during the pandemic (*P* = 0.007). The higher severity of DKA (0.026) and the need for higher doses of insulin (*P* = 0.005) were also observed. The hospitalization rate was higher during the corona peaks, particularly peaks 1 and 4, compared to the non-peak days of COVID-19.

**Conclusion:**

The increase in the incidence of diabetes (new cases) in the pandemic can suggest the role of the COVID-19 virus as an igniter. Also, as a trigger for the higher incidence of DKA with higher severities, which is probably caused by more damage to the pancreatic beta cells and requires higher doses of insulin.

## Introduction

The role of some viral infections such as congenital rubella in the occurrence of type 1 diabetes in children has been confirmed.

Various autoimmune complications have been reported in the COVID-19 epidemic, such as neurological, rheumatological, cardiovascular, skin complications, and even type 1 diabetes and diabetic ketoacidosis in children and adolescents [1].

Considering that few studies have been reported on type 2 diabetes in hospitalized adults. The Viola study reported more severe diabetes and DKA during the COVID-19 pandemic in Italian type 1 diabetes patients in 2020.The binding of SARS COV2 to ACE2 receptors in tissues and metabolic organs such as pancreatic beta cells, kidney, intestinal adipocytes can become a factor in triggering the mechanism of type 1 diabetes in patients. She suggested that the corona pandemic will probably trigger the occurrence of T1DM in the future [2].

In R. Allard’s study of T1DM patients during the 2009 H1N1 pandemic, diabetes clearly aggravated the severity of infection, and uncontrolled blood sugar index clearly increased mortality [3]. In Suheda’s study, the possible mechanisms of the exacerbation of the COVID-19 infection in diabetic patients are described as follows: In diabetic patients, an increase was seen in ACE2 receptors, which is favored by SARS-COV-2. In addition, an inappropriate immune response caused by the function of macrophages and monocytes, which intensifies the inflammatory cycle and causes more damage of cytokines to the lung was raised. Also, alveolar dysfunction is seen more often in diabetic patients which disturbs ventilation [4].

On the other hand, coagulation disorders in type 2 diabetes patients in the form of hypercoagulability, increase in fibrinolysis markers and platelet activity and attachment to vascular endothelium have been reported [4].

Xiaofan Jia’s 2021 study found no difference between the prevalence of SARSCOV2 antibodies in children and adults with and without diabetes in Colorado, and no evidence of an increased risk of COVID-19 in children and adolescents with type 1 diabetes (T1DM) was reported [5]. In Richardson’s study in New York hospitals in 2020, 33.8% of hospitalized patients had Type 2 diabetes, which is one of the most common comorbidities [6].

In the studies of M.C.G. Wuz and F. Zhou, which are related to 2020, the mortality of corona patients with type 2 diabetes was reported to be about 3 times that of the general population, and the prevalence of mortality of diabetic patients with Middle East respiratory syndrome coronavirus (MERS COV) in X Yang’s study was 50% compared to the general population, which was about 20% [7–9]. In the Holman’s cohort study in England, the risk of mortality related to Covid-19 in type 1 and type 2 diabetes patients was not related to the severity of hyperglycemia, unlike Suheda’s study, where comorbidity was more common in diabetic patients with corona, and with better blood sugar control, the prognosis of patients was also better [10]. In the 2020 Y Pan’s study, fever and cough were reported as the most common symptoms of hospitalized diabetes patients with COVID-19 [11]. In Wang Aihony’s 2020 study, improper blood sugar control reduced the lymphocyte proliferative response compared to neutrophil and macrophage function, and corona infection was introduced as a stressful and inflammatory factor that predisposes diabetic patients to DKA [12]. A meta-analysis of 12 studies by Fadini and his colleagues showed that overall, diabetes does not increase the risk of Covid-19 infection, but infected patients have a more severe disease and a worse prognosis [13]. In the summary of 15 studies by Mahmoud Nassar and his colleagues in 2021, the prevalence of T1DM in children and adults with COVID-19 was 0.15–28.98%. The. rate of positive COVID-19 PCR in diabetic patients was reported to be 16.68%, the most common symptoms of which were cough, vomiting, runny nose, fever, hyperglycemia, and even DKA [14]. In OA Ebekozien’s study, 15.6% of 33 hospitalized diabetic patients with COVID-19 infection were newly diagnosed with diabetes [15]. Unsworth’s study during the Corona era showed that 90% of the 33 hospitalized patients were new cases of type 1diabetes, and only 5 of them were positive for COVID-19 PCR [16]. In the I Rabbone’s study, despite the reduction of new cases of T1DM in 2020 compared to 2019 in Italy, severe DKA cases were seen more in these patients, which was probably due to the delay in visiting hospitals [17]. Akhtar Hussain’s study showed that better glycemic index control is helpful in managing T2DM patients with COVID-19 infection and old age and diabetes were risk factors for higher severity of corona disease. Whether diabetes itself increases the risk of infection, morbidity and mortality in these patients or these conditions are caused by microvascular and macrovascular complications of diabetes is still unknown [18].

This study was conducted with the aim of understanding how COVID-19 acts on type 1 diabetes and DKA and better management in treatment of children and adolescents affected by the national corona epidemic and prevention of late diagnosis and treatment.

## Methods

This was a cross-sectional descriptive-analytical study. Our study began at the end of the first year of the Covid-19 pandemic and the observation of frequent cases of hospitalization of patients with type 1 diabetes and DKA state. The files of all children and adolescents with type 1 diabetes hospitalized in Children’s Referral of Amirkabir Hospital in Markazi Province two years before and two years after the start of the pandemic of COVID-19, including six corona peaks in the country were investigated (January 2018–2022).

The desired variables including the frequency of hospitalized patients (known and new cases), frequency of DKA, severity of DKA, duration of recovery from DKA status, age, body mass index, duration of hospitalization, clinical symptoms including cerebral edema, laboratory indexes, acute kidney injury and the total amount of daily insulin required at the time of discharge were compared and statistically analyzed.

Determining the peaks was based on the graph and in the places where the peaks of the waves were located and, in order to be more objective, it was defined based on the higher daily incidence of definite cases (based on the PCR test) of COVID-19 out of 100 people (average monthly). Of course, this definition was possible for the third to sixth waves, and for the first and second waves, when the testing was done much less due to the limitations of the kit and there were more defects in the diagnosis of Covid-19 cases, it was inevitably done only based on the provincial chart and comparing it with the diagram of the entire country.

The severity of DKA is defined by the degree of acidosis: mild, venous pH 7.2 to 7.3 or serum bicarbonate 10 to15 mmol/L; moderate, pH 7.1 to 7.2 or serum bicarbonate 5 to less than 10 mmol/L; severe, pH under 7.1 or serum bicarbonate less than 5 mmol/L. The normal corrected sodium based on blood glucose was defined 135_145 mmol/L [19, 20]. 

Acute kidney injury (AKI) has been traditionally defined as an increase in serum creatinine by ≥ 0.3 mg/dL from baseline within 48 h; or an increase in serum creatinine to ≥ 1.5 times baseline within the prior 7 days; or Urine volume ≤ 0.5 mL/kg/hour for 6 h [20].

Cerebral edema criteria include headache and slowing of heart rate, change in neurological status (restlessness, irritability, increased drowsiness, and incontinence), specific neurological signs (e.g., cranial nerve palsies, papilledema), rising blood pressure, and decreased O2 saturation [21, 22].

After entering the data into the statistical software (SPSS version 22), frequency, percentage, mean and standard deviation were used to describe the data. Data analysis was done using chi-square, t-test or their non-parametric equivalents. The significance level was considered 0.05 in the analytical analysis.

## Results

**Fig. 1:**
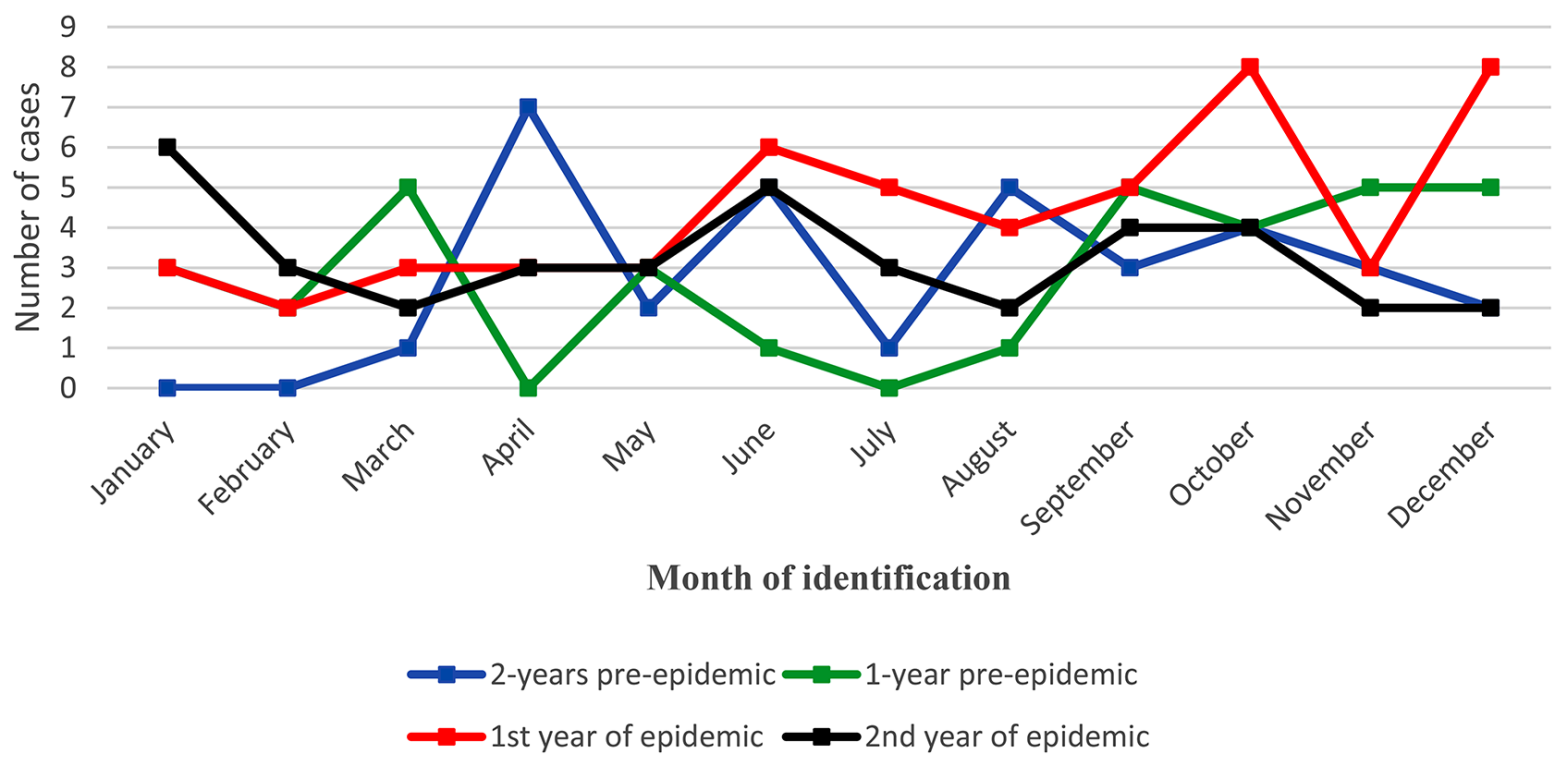
Newly identified cases of type 1 diabetes by year, during 4 years of evaluation

In a 4-year period including two years before the start of the COVID-19 pandemic and two years after the observation of the first case of COVID-19, information on all cases of type 1 diabetes(T1DM) in children under 18 years of age in Arak was recorded with no missing data. In general, there were 334 cases of visits to the hospital, which in the two years before the COVID-19 pandemic, the number of presentations per month was equal to 6.58, and in the two years of the pandemic, it was equal to 7.33 cases per month (Table 1). Of these, 159 cases were new cases of T1DM, of which 67 cases were related to two years before the start of the pandemic and 92 cases were related to two years of the pandemic, the difference of which is statistically significant (*p* = 0.006). In addition, the most of new cases (53 cases) were identified in the first year of the pandemic period (Table 1; Fig. 1). The comparison between the new cases in the first year of COVID-19 and those in the second year was not significant (*p* = 0.14) (Table 1; Fig. 1).

**Table 1:** Comparison of demographic and therapeutic characteristics of T1DM patients before and during Covid-19 epidemic

Characteristic	Categories	Pre- pandemic	Pandemic	*P*-Value
Total admission	Frequency	151(6.58 per month)	183(7.33 per month)	0.08
New cases	Frequency	62(2.69 per month)	97(3.88 per month)	**0.006**
Age	Mean	9.35	9.04	0.50
Age of new cases	Mean	8.56	7.87	0.30
Sex(male)	Male	82(54.3%)	94(51.4%)	0.68
	Female	69(45.7%)	89(48.6%)	
DKA	Frequency	61(40.4%)	101(55.2%)	**0.007**
DKA severity	Non-DKA	90(59.6%)	82(44.8%)	0.026
	Mild to moderate	22(14.6%)	38(20.8%)	
	Severe	39(25.8%)	63(34.4%)	
Recovery from DKA	Day	1.54	1.69	0.36
BMI	kg/m2	19.96	18.45	0.14
Insulin dose	Unit	31.82	34.56	0.20
Insulin dose	U/Kg/d	0.93	1.07	**0.005**
ICU time	Day	1.82	1.31	0.13

**Fig. 2:**
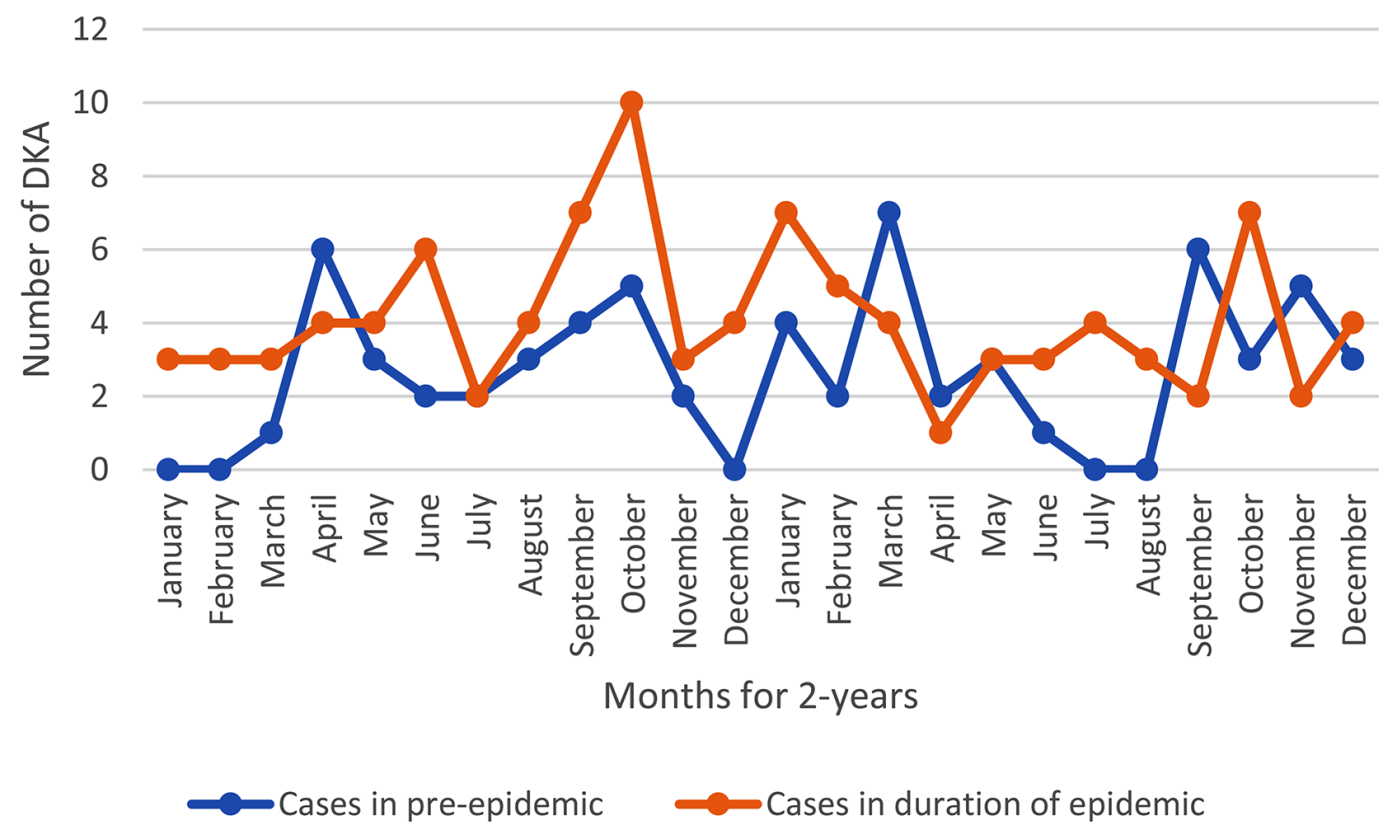
Case of DKA hospitalization before and during Covid_19 pandemic in T1DM

**Fig. 3:**
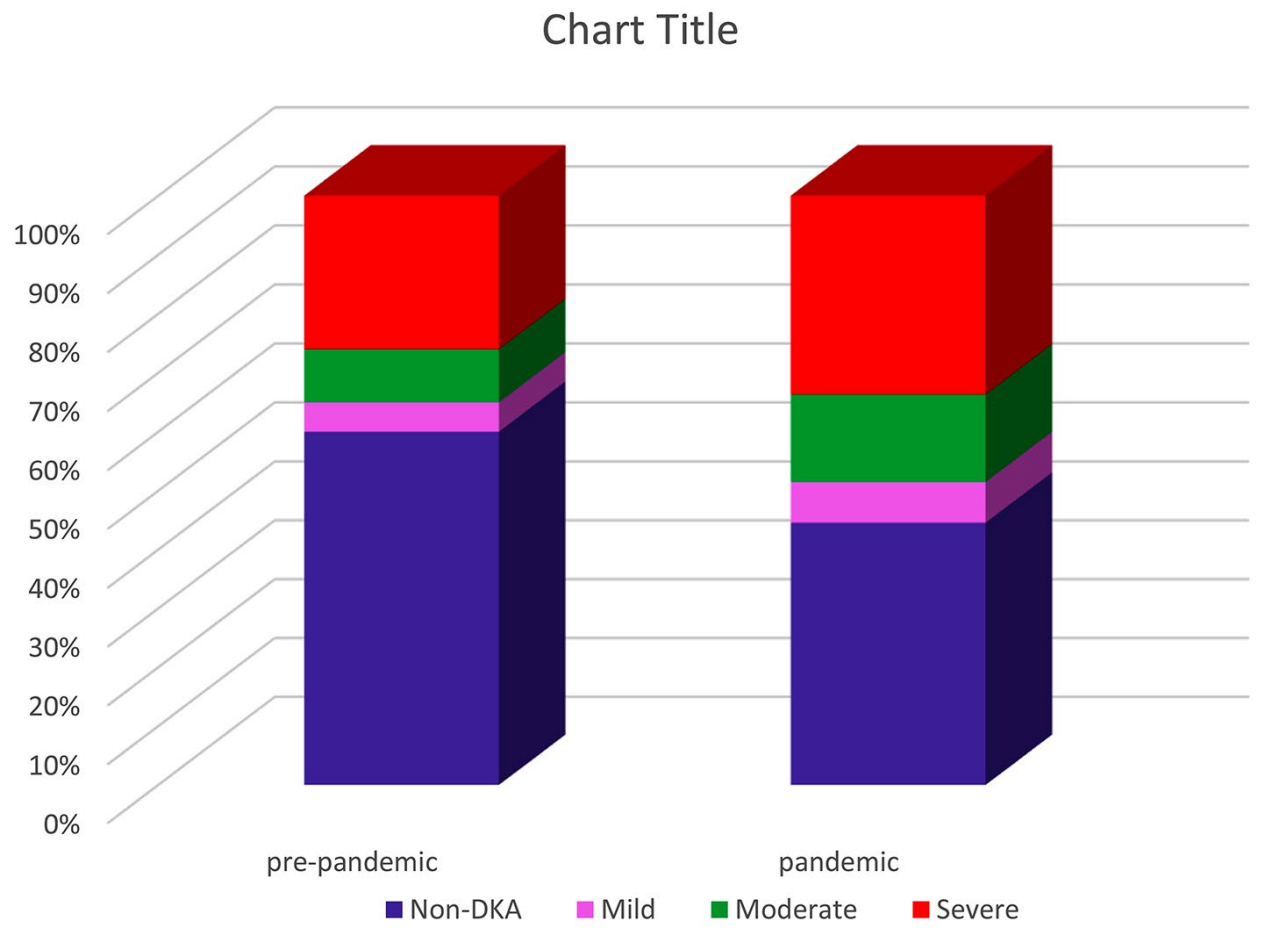
Comparison of DKA severity before and during Covid_19 pandemic

In this study, the age range of the patients was from 1 to 18 years and their average age (standard deviation) was 9.18 (4.16) years; both the average age of all patients and that of new patients in the pandemic period were lower than the pre-pandemic period, though this difference was not statistically significant. About 53% of the investigated patients were boys, and this ratio was not significantly different during the epidemic period and before that (Table 1). In the two years before the COVID-19 pandemic, there were 61 cases and in the two years of the pandemic, there were 101 cases of DKA presentation, where the difference was statistically significant (*p* = 0.007) and the odds ratio was 1.82 (95% CI 1.17, 2.81) (Table 1; Fig. 2). In addition, the severity of DKA states were significantly higher during the pandemic period (*p* = 0.026) (Table 1; Fig. 3).

**Fig. 4:**
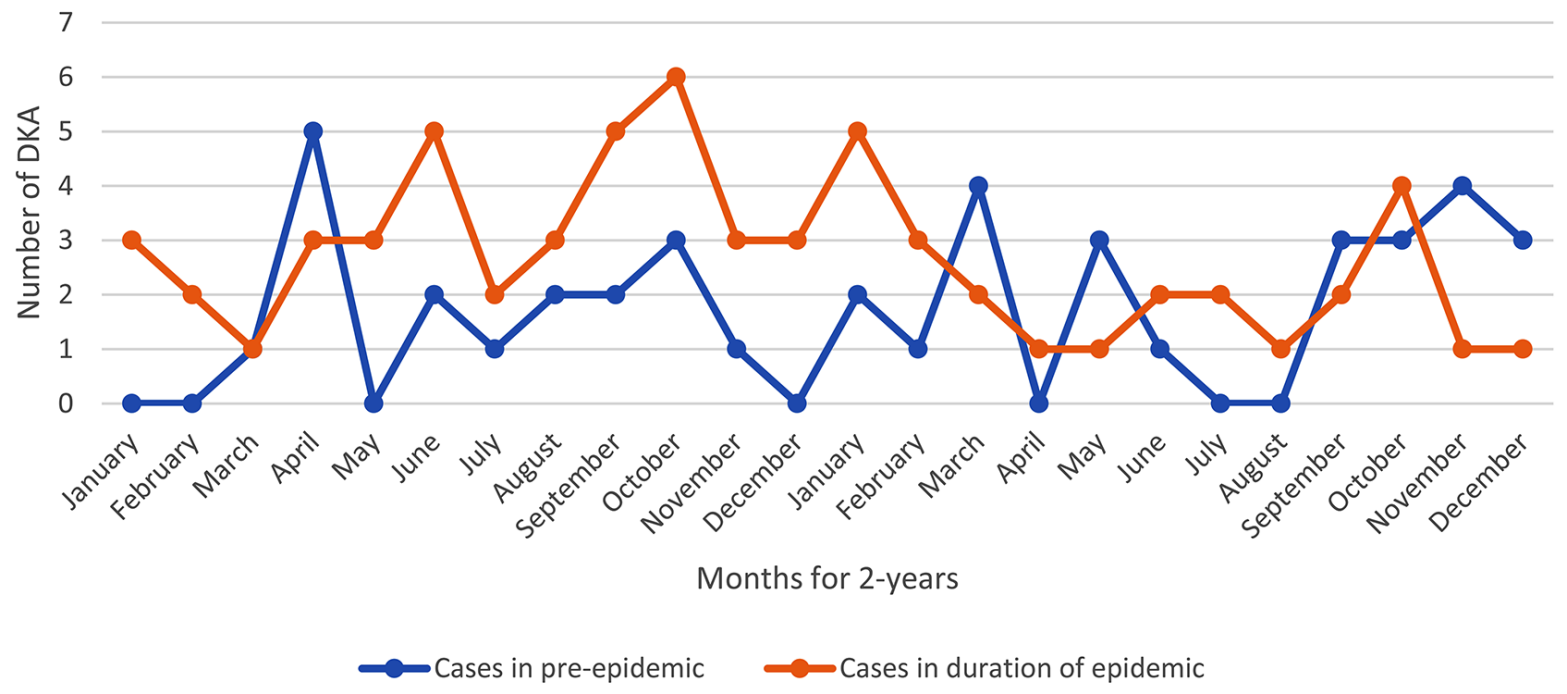
Manifestations of DKA in newly diagnosed T1DM before and during Covid_19 pandemic

The first manifestation of newly diagnosed T1DM patients as DKA during hospitalization before COVID-19 was 61.2% (41 case) and 69.6% (64 case) during the pandemic (Fig. 4).

**Fig. 5:**
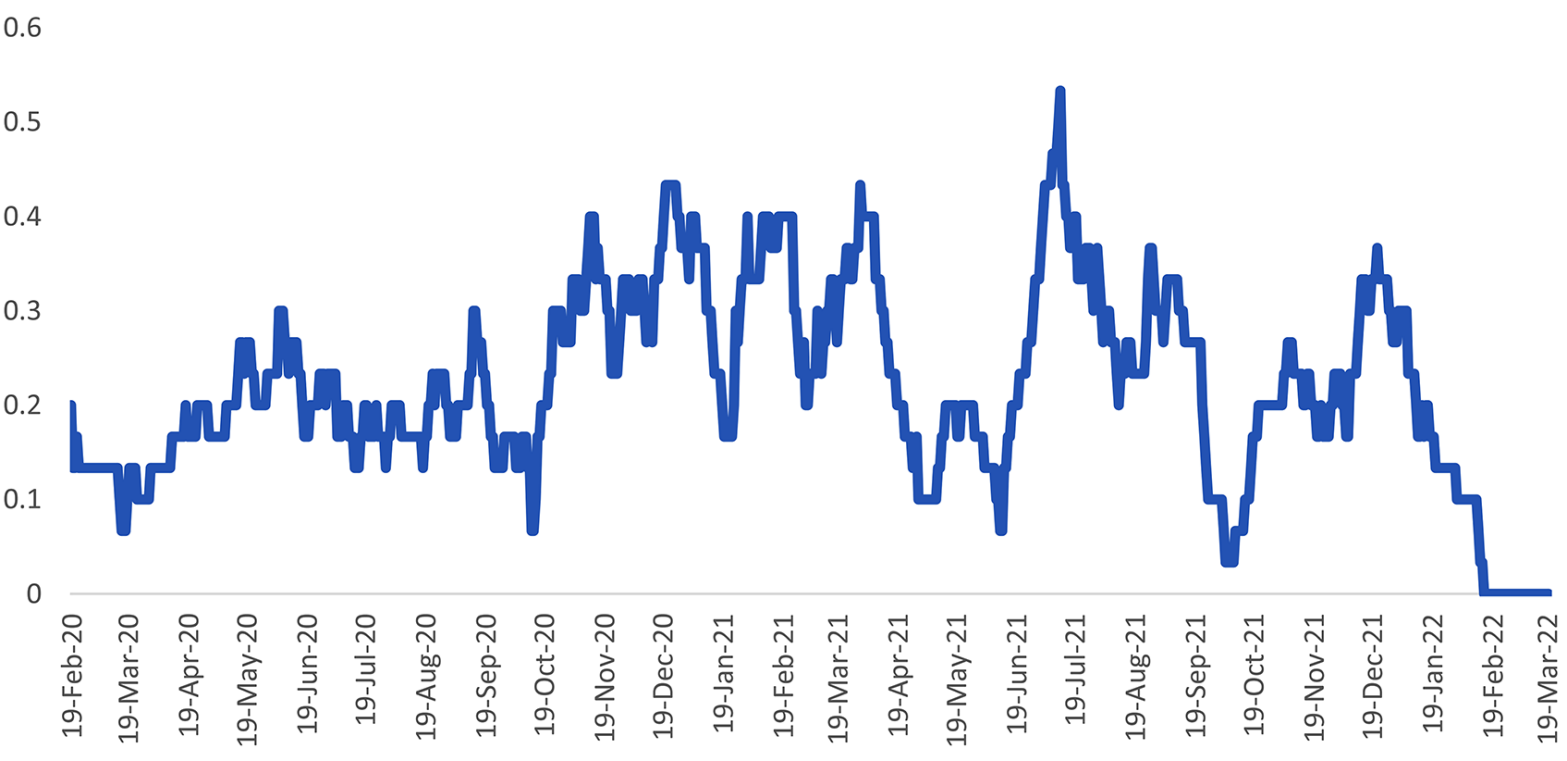
The trend of changes in hospitalization of T1DM during the Covid_19 pandemic in Amirkabir Hospital

In terms of the duration of exit discharge from ketoacidosis during the pandemic, this period was longer than that before the pandemic, but the difference was not statistically significant (Table 1). Total T1DM admission increased significantly during the peaks (0.037), but the number of new cases did not show a significant increase. The highest number of new T1DM cases per month was observed in peaks 1, 4, 3,5,6 and 2 respectively (Table 2; Fig. 5). There was no significant difference in age, patient gender, severity of DKA, patients’ laboratory findings, length of stay in PICU and insulin dosage at the time of discharge in corona peaks and days between peaks (Table 2).

**Table 2:** Comparison of demographic and therapeutic characteristics of T1DM patients at the peaks of Covid_19 and between them

Characteristic	Categories	Peaks	Between peaks	*P*-Value
Total admission	Frequency	97(8.41 per month)	86(6.19 per month)	**0.037**
New cases	Frequency	51 (4.42 per month)	46(3.3 per month)	0.15
Age	Mean	8.84	9.28	0.48
Age of new cases	Mean	7.22	8.59	0.09
Sex(male)	Male	48(49.5%)	46(46.5%)	0.59
	Female	49(51.5%)	40(53.5%)	
DKA	Frequency	47(48.5%)	54(62.7%)	0.052
DKA severity	Non-DKA	50(51.5%)	32(37.2%)	0.13
	Mild to moderate	14(14.4%)	24(27.9%)	
	Severe	33(34.1%)	30(34.9%)	
Exit from DKA	Day	1.71	1.68	0.89
BMI	kg/m2	18.02	18.93	0.39
Insulin dose	Unit	34.13	35.03	0.76
Insulin dose	U/Kg/d	1.07	1.07	0.93
ICU time	Day	1.32	1.30	0.95

In total, seven people diagnosed with cerebral edema received mannitol during DKA attacks, one of whom was related to the pre-corona period, and the rest were related to the Corona period and peak days (two patients in the third peak, two in the fifth peak, and two in the sixth peak).

In total, 15 patients had acute kidney injury during DKA attacks, six of whom were related to two years before Corona and nine were related to two years of Corona. Out of these nine people, six patients were related to peak days and three were related to the time between the peaks. No special difference was observed in the clinical manifestation and laboratory data of hospitalized patients both before and during the corona period (Tables 3 and 4). The total daily dose of insulin needed by patients at the time of discharge (IU/Kg) during the pandemic period was clearly higher than that by patients before the pandemic period (Table 1).

All patients have records and are under insulin therapy and regular examinations. And fortunately, none of them have suffered long-term complications after corona and diabetes. 2 obese prediabetic girls (16 and 18 years old) with a history of polycystic ovary syndrome who were treated with metformin and entered the DKA phase following corona infection. Fortunately, after 3 months of discharge, Insulin was gradually discontinued and now treated with Metformin. Of course, these 2 patients were excluded from the list of statistical analysis because they were not classified as type 1 diabetes.

**Table 3:** Symptoms of patients with type 1 diabetes before and during COVID_19 pandemic

Symptom	Pre- pandemic (%)	Pandemic (%)
Pulmonary	7.9	12.6
Digestive	45	47
Neurology	17.2	20.8
Urinary	55.6	65.6
Fever	9.3	8.7
Cough	1.3	4.4
Other	27.8	27.3

**Table 4:** Comparison of laboratory characteristics of children with type 1 diabetes before and during COVID_19 pandemic

Lab Data		Pre- pandemic	Pandemic	*P*-Value
WBC		11.5	12	0.58
RBC		5.12	5.15	0.62
ESR		11.26	10.13	0.54
K		4.18	4.25	0.29
NUT		57.56	55.39	0.33
HGB		14.04	14.13	0.64
Urea		28.68	29.99	0.32
LYMPH		35.9	37.86	0.39
PLT		301.44	309.41	0.49
Cr		0.94	0.94	0.93
Na	Hyponatremia	35	56	0.12
	Normal	69	91	
	Hypernatremia	6	2	
CRP	Negative	125	119	0.12
	Plus1	4	14	
	Plus2	4	4	
	Plus3	4	3	

## Discussion

For a long time, viral infections have been considered as a risk factor for Type 1 DM in infants and children, and only the relevance of Congenital rubella infection has been confirmed so far [19, 20]. However, coxsackievirus has been mentioned in some studies with the possibility of causing chronic prediabetic period and increasing HbA1C [23]. The increase of anti-islet antibodies following rotavirus and enterovirus infections has been mentioned in conflicting reports [24]. Increased production of interleukin 2 in the inflammatory processes can be a pathological factor in causing damage to pancreas beta cells [25].

Of 334 type 1 diabetic patients hospitalized during for four years, the total hospitalization rate of patients including new and known cases before and after the pandemic was not significantly different, although the total of cases hospitalized in the first year of the pandemic was slightly higher than that in the second year. New cases of T1DM were significantly more during the pandemic period (*P* = 0.006), which means more incidence of T1DM during the pandemic (Table 1). This finding was like that of the study of Bryne [26], Clemens [27] and contrary to the results reported by Xiaofan [5], Fadini [13], Josephine [28], Catherine [29], Lawrence [30], Aysun [31].

Considering that, the longest monitoring time (study) and the largest number of children with type 1 diabetes during the pandemic is related to the present study, this finding is very important. Because the covid-19 virus can be considered as a factor in the flare-up and occurrence of type 1 diabetes. On the other hand, pathophysiological evaluations such as similar biological markers in pancreatic beta cell receptors and covid-19 virus and how to activate autoantibodies that cause type 1 diabetes can be very helpful and useful. What is certain is that type 1 diabetes patients have a genetic predisposition to the disease, and the flare-up of inflammatory factors following some infections can cause the disease to occur earlier and more severely. And perhaps the covid-19 virus can also be added among the notorious viruses that cause type 1 diabetes, such as congenital rubella.

Of course, multicenter studies (which we are doing) should be conducted with different races regarding the increase in the incidence of type 1 diabetes in children. Since the severity, mortality and complications caused by the corona virus were completely different in different peaks and in different countries (with different races) [32].

The reason why the total number of hospitalized T1DM patients was not significantly different in the pre- and post-pandemic period was related to the hospitalization of known cases of T1DM patients with poor control, which was aimed at regulating blood sugar, adjusting insulin dosage, and re-educating them in the pre-pandemic period. During the pandemic, the fear of COVID-19 infection in the hospital prevented physicians from approving hospitalization and patients from being hospitalized due to poor control, so most of the patients during the pandemic were new cases of T1DM. Because in the pre-pandemic era, patients with poor glycemic control tended to be hospitalized for insulin dose adjustment and further education. Therefore, it can be stated that the incidence of diabetes (new cases) has clearly increased during the COVID-19 pandemic (Table 1; Fig. 1).

The average age of hospitalized patients during the four years of the study was about nine years, which was a little lower during the pandemic, particularly for new identified cases, though it was not statistically significant, as was reported in M. Sinéad’s study [33]. No gender difference was observed in diabetic patients both before and during the pandemic, and even during the peaks of pandemic in hospitalized patients, which can indicate that immunological vulnerability in inflammation caused by corona virus does not depend on gender (Table 1).

Obesity is a major risk factor for contracting COVID-19 infection and its complications, resulting in higher mortality rates due to cytokine storm and coagulopathy [34]. Also, obesity-induced chronic inflammation and disruptions of insulin and leptin signaling can result a disproportionate or hyper-inflammatory response, which, together with elevated ferritin levels, can be a direct cause for ARDS and cytokine storm. However, there was no difference in Body Mass Index (BMI) in type 1 diabetic patients before and during the COVID-19 pandemic (Table 1). Obesity was not a risk factor for type 1 diabetes despite the pathophysiology’s suggested for cytokine flares in obese patients with COVID-19 infection.

Out of 162 DKA patients, 101 were admitted to PICU during the COVID-19 pandemic, which was clearly higher than that before the pandemic (*P* = 0.007) and odds ratio of 1.82 (95% CI 1.17, 2.81) (Table 1; Figs. 2 and 4). Similar findings were reported in other studies [28–31, 35]. Also, the severity of DKA was higher during the COVID-19 pandemic (*P* = 0.026) like Rabbone’s results [16] (Table 2; Fig. 3). This can be caused by patients’ late visits to the hospital due to the fear of being hospitalized during the corona period and even the delay in early diagnosis of the patient due to the attribution of clinical symptoms of DKA (abdominal pain, nausea, vomiting, dyspnea and so) to manifestations of viral infection. Of course, the possibility of accelerating the destruction of pancreas beta cells following COVID-19 infection through inflammatory factors as trigger factors and autoimmune reactions for the occurrence of DKA should also be considered [23, 24, 35].

The duration of recovery from DKA was longer in hospitalized patients during the pandemic period, which can be justified by considering the greater number and severity of DKA.

Interestingly, the amount of insulin needed by patients at the time of discharge (IU/Kg) during the pandemic period was clearly higher than that by patients before the pandemic period (Table 1), which could be due to the greater destruction of pancreatic beta cells due to inflammatory conditions, as well as the recovery from DKA, when the need for higher doses is obvious compared to non-DKA patients, (the outbreak of DKA was higher during the corona period) [20].

The hospitalization rate of patients was clearly higher during the corona peaks because infections are the triggering factor for DKA. Although the number of new cases did not show a significant increase in the 6 national corona virus peaks during the two years of the pandemic, the most newly identified T1DM cases were observed in peaks 1, 4, and 3, respectively. This may be justified based on the mutations of the virus and the exposure of the immune system to new strains and the destruction of beta cells of the pancreas in the peaks of the pandemic, especially in patients who suffered from autoimmune diseases [1].

There was no significant difference in age, patient gender, severity of DKA, patients’ laboratory findings, length of stay in PICU and insulin dosage at the time of discharge in corona peaks and days between peaks (Table 2).

The most common clinical symptoms of patients during the COVID-19 pandemic were polyuria, nausea and abdominal pain, which showed no difference from the pre-corona period (Table 3). However, since these symptoms were interpreted based on corona infection, unfortunately, more patients were referred to the hospital with delay and in DKA state.

No special difference was observed in the laboratory data of hospitalized patients both before and during the corona period. (Table 4)

Out of seven patients with brain edema symptoms (according to the criteria) who were treated with mannitol, six were during the COVID-19 pandemic, which could be justified due to the severity of DKA, especially because they had disturbed electrolytes at the beginning of hospitalization. Fortunately, all are now under diabetes control without sequel. There were 15 hospitalized DKA patients, of which 9 were during the COVID-19 pandemic, which were diagnosed with acute kidney injury (according to the criteria) and received dopamine and pediatric nephrology consultations. There was no significant increase of acute kidney injury (according to the criteria) during DKA state, pre and during Covid pandemic.

The limitations of this study included the lack of diagnostic kits for COVID-19 in the first year of the pandemic. On the other hand, a more comprehensive study in more ethnically diverse populations could be performed in a multicenter manner and provide more valuable information to judge the behavior of COVID-19 in the incidence and prevalence of T1DM. In conclusion new cases of T1DM were significantly higher during the pandemic period, which means more incidence of T1DM during the pandemic. Therefore, it can be stated that the incidence of diabetes (new cases) has clearly increased during the COVID-19 pandemic. This can indicate an etiological role of the COVID-19 virus. Age, sex, BMI, clinical manifestations, laboratory data and total admissions in type 1 diabetic patients were not observed before and during the pandemic, even the picks and between them. DKA and severity of DKA were clearly higher than before the pandemic. Interestingly, the total daily insulin dose required by patients at discharge (IU/Kg) during the pandemic period was clearly higher than the pre pandemic period, which could be due to the greater destruction of pancreatic beta cells due to inflammatory conditions, as well as higher severity of DKA, when the need for higher doses is obvious compared to non-DKA patients. It seems that public education and more attention of physicians to the general symptoms of diabetes and DKA, especially in children and adolescents in viral epidemics, it can be useful in timely diagnosis and treatment and prevention of morbidity and mortality due to high severity of DKA.

## Data Availability

All data generated or analyzed during this study will be available via contacting the corresponding author.

## Abbreviations

T1DM: Type 1 Diabetes
DKA: Diabetic Ketoacidosis
AKI: Acute Kidney Injury
PICU: Pediatric Intensive Care Unit
